# The effect of simvastatin–*Acalypha indica* Linn. combination on the improvement of fatty pancreas in rats induced with a high fructose and cholesterol diet

**DOI:** 10.5455/javar.2022.i601

**Published:** 2022-06-30

**Authors:** Desak Gede Budi Krisnamurti, Siti Farida, Rizky Clarinta Putri, Wilzar Fachri, Erni Hernawati Purwaningsih

**Affiliations:** 1 Department of Medical Pharmacy, Faculty of Medicine, Universitas Indonesia, Jakarta, Indonesia; 2 Drug Development Research Cluster, Indonesia Medical Education and Research Institute (IMERI), Faculty of Medicine, Universitas Indonesia, Jakarta, Indonesia

**Keywords:** *Acalypha indica* Linn., cholesterol, fatty pancreas, fructose, simvastatin

## Abstract

**Objective:**

The study aimed to determine the effect of *Acalypha indica* Linn. (AI) root extract and a combination of simvastatin–AI on improving the fatty pancreas in Sprague–Dawley rats induced with a high fructose and cholesterol diet.

**Materials and Methods:**

Twenty-four male Sprague–Dawley rats were induced with a high fructose and cholesterol diet for 4 weeks before being divided into four groups. Each group receiving treatments consisting of simvastatin only, *A. indica* extracts only, or simvastatin–*A. indica* extract combination. A histological examination was conducted to determine the effect of each treatment. Also, one-way analysis of variance (ANOVA) and *post-hoc* Bonferroni test were conducted to assess the comparison of groups from the histological examination.

**Results:**

Significant improvement was found in fatty pancreas between rats without therapy and rats treated with simvastatin therapy (*p* = 0.024, 95% CI: 0.038–0.696), and also between rats without treatment and rats treated with simvastatin–*A. indica* extract combination therapy (*p* = 0.000, 95% CI: 0.241–0.873) using one-way ANOVA and the *post-hoc* Bonferroni test.

**Conclusions:**

The results of the combination of simvastatin–*A. indica* Linn. root extracts treatment showed a synergistic effect on the improvement of fatty pancreas, but further research is needed to find potential adverse effects on the interaction of these two substrates to confirm the safe use of this treatment.

## Introduction

Changes have occurred in people’s trends of daily nutritional intake. Fructose is now more frequently used in daily foods, especially bottled beverages with sweetened corn syrup. In America, fructose accounts for up to 46% of the beverages with sweeteners [1]. In addition, there has also been an increase in cholesterol intake, mainly from red meat, processed meats, and eggs. This high-fructose, high-cholesterol diet raises the risk of obesity, cardiovascular disease, and non-insulin-dependent diabetes [1–5].

The above-mentioned conditions lead to metabolic syndrome. Metabolic syndrome and obesity have been strongly associated with nonalcoholic fatty pancreas disease (NAFPD) [6]. One study in Jakarta showed that the prevalence of NAFPD found during a medical checkup examination reached up to 35% [7]. A high-fructose and high-cholesterol diet is an indirect cause of NAFPD because it leads to obesity, insulin resistance, diabetes, and metabolic syndrome, all strongly associated with NAFPD [8].

The main principle of managing NAFPD is to address its underlying cause. Management consists of non-pharmacological management, such as lifestyle modification. Even though there is no approved pharmacological therapy, consuming lipid-controlling drugs, such as fibrate, and statins, such as simvastatin, can reduce the symptoms [9]. In previous research, AI extract was found to have antidiabetic and anti-hyperlipidemia effects that could potentially have a role in managing NAFPD [10]. However, no studies have yet examined the effects of AI extract with a combination of simvastatin on improving fatty pancreas. So, the goal of this study was to find out what happens to the fatty pancreas in rats fed a high-fructose and high-cholesterol diet when simvastatin and *A. indica *extract is given together.

## Materials and Methods

### Ethical approval

The experimental procedures were approved by the Ethics Committee of the Faculty of Medicine, University of Indonesia, with ethical approval number 19-06-0660. 

### Extract preparation

The AI samples were gathered from Depok, West Java, Indonesia, and their identity was verified by the Indonesian Institute of Sciences (LIPI) in Bogor. The root was dried, powdered, and then macerated using 70% ethanol for 24 h. After macerating the residue three times, liquid extracts were concentrated to semisolid using a rotavapor. The extract was diluted with distilled water and administered to experimental animals.

### Animal preparation

This study used a total of 24 male Sprague–Dawley rats. The induction of Sprague–Dawley rats used a diet high in fructose and cholesterol for 4 weeks. The rats were grouped into four therapeutic groups, which consisted of a negative control group (distilled water only), and three positive control groups (10 mg/kg BW simvastatin; 250 mg/kg BW AI root extract; and a combination therapy of 10 mg/kg BW simvastatin and 250 mg/kg BW AI). After 4 weeks, the pancreases of the rats were used for histological examination.

### Histological examination

The rats’ pancreases were soaked for 24 hours in 10% formaldehyde, cut longitudinally, and then dehydrated gradually using 70%, 80%, 90%, and 100% alcohol. Clearing of the pancreas tissue used xylol 1 and 2 solutions, followed by histotechniques of pancreas tissue using paraffin blocks and hematoxylin–eosin staining. For the calculation, the areas of fat cells and normal pancreas cells are used, and the fibrotic area in five fields of view for each histology slide of the pancreas is used to determine if a rat has a fatty pancreas.

### Data analysis

The ImageJ program v.1.6.0 was used to calculate the percentage of fat cell area and normal pancreatic cells. The histological examination results were analyzed using one-way analysis of variance (ANOVA) and *post hoc* Bonferroni test.

## Results and Discussion

### Fatty pancreas images in rats

**Figure 1.**
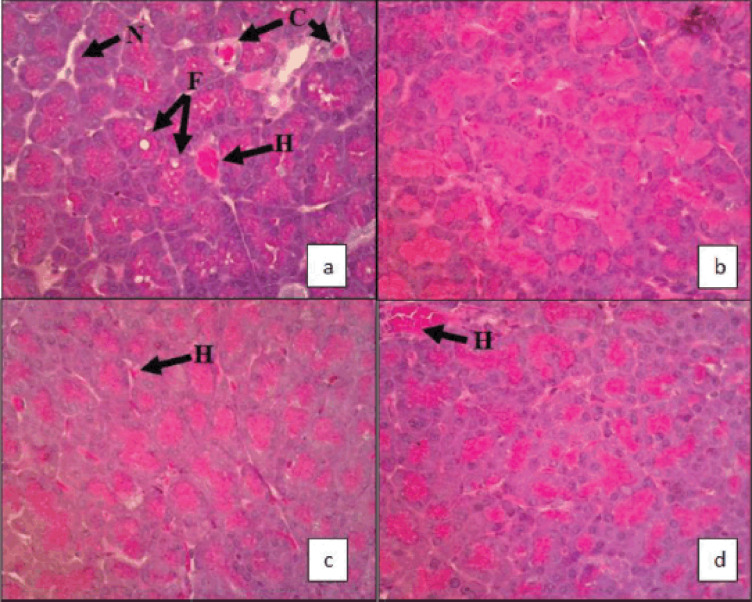
Histopathological features of a rat’s pancreas after being induced with a high fructose and cholesterol diet. (a) Control group showing congestion (C), fat infiltration (F), hemorrhage (H), and necrosis (N). (b) Simvastatin therapy group showing reduced infiltration of adipocyte cells on pancreas cells, and the percentage of fat cells compared to pancreas cells is 0.272%. (c) *Acalypha indica *extract therapy group showing reduced infiltration of adipocyte cells on pancreas cells with a percentage of fat cells compared to pancreas cells, which is 0.310%. (d) Simvastatin–*A. indica* extract combination therapy group showing reduced infiltration of adipocyte cells on pancreas cells and the percentage of fat cells compared to pancreas cells is 0.178%.

The pancreases of rats were examined under a 400× magnification microscope in five different fields of view (Fig. 1). The fatty pancreas was evident in the histopathological features of the control group. From Figure 1, we can see the infiltration of adipocyte cells in the fatty pancreas in the control group that was consistent with those described in previous studies [11]. There is not yet a theory that has been widely accepted on NAFPD. Still, the infiltration of adipocyte cells into pancreatic cells shows a strong association with obesity and non-insulin-dependent diabetes mellitus [12]. Furthermore, the major risk factors for NAFPD are metabolic syndrome, dyslipidemia, insulin resistance, and low serum lipase activity. Consuming a diet high in fructose and cholesterol can trigger the risk factors mentioned above, causing it to become an indirect risk of NAFPD [7,11]. 

In this study, no fibrosis was found in the histopathological features of the pancreas. This differs from previous studies and can be caused by the duration of the high fructose and cholesterol diet given to the rats in this study, which was only shown for 4 weeks, shorter than previous studies which found fibrosis in NAFPD, in which the diet given was for 6–18 weeks [13]. 

### Histopathology result of the rats’ pancreases

Table 1 shows the results of the calculations of the fatty pancreas in rats. The median percentage of fat cells compared to the overall pancreatic cells in each group was 0.247% (0.145%–1.014%).

One-way ANOVA showed *p* < 0.05, which concluded at least two groups with the percentage of the fatty pancreas were significantly different. The analysis was then continued with a *post-hoc* Bonferroni test to compare groups with a significantly different fatty pancreas percentage. Results from the *post-hoc *analysis showed a statistically significant difference in fatty pancreas between control groups and the simvastatin therapy group with *p* = 0.024 (95% CI: 0.038–0.696) and between control groups with simvastatin–AI extract combination therapy with *p* = 0.000 (95% CI: 0.241–0.873).

There is no previous study on the effects of simvastatin on NAFPD. Still, several studies have mentioned the relationship between simvastatin and the increased survival rate of patients with pancreatic cancer [14,15]. The simvastatin treatment, in addition to being one of the preferred therapies in the management of NAFLD [6], is said to be related to NAFPD [9,16] and can decrease low-density lipoprotein (LDL), cholesterol, and triglyceride levels in the blood, so theoretically, it can decrease lipid deposition in adipocyte tissues, which in excess conditions causes obesity. The study showed a statistically significant change in the percentage of the fatty pancreas in experimental animals before and after administering AI extract and simvastatin. From histological findings, congestion, fat infiltration, and necrosis cannot be found in the group treated with the simvastatin–AI extract. Improvement of the fatty pancreas followed the theory and study results, which showed the effects of simvastatin in treating pancreatic disorders. However, further research is still needed to confirm its clinical significance.

**Table 1.** Calculation results of the fatty pancreases in rats. The median percentage of fat cells compared to the overall pancreas cells in each group was 0.247% (0.145%–1.014%).

Group	Percentage of fat cells compared to pancreas cells
Control (high fructose and cholesterol diet)	0.699%
Simvastatin (high fructose and cholesterol diet with simvastatin therapy)	0.272%
*A. indica *extract (high fructose and cholesterol diet with *A. indica *extract therapy)	0.310%
Simvastatin–*A. indica *extract combination (high fructose and cholesterol diet with simvastatin–*A. indica *extract therapy)	0.178%

In a previous study on rats, AI extract was known to have antidiabetic and anti-hyperlipidemic effects. Flavonoids and polyphenols in *A. indica *can reduce LDL and increase serum levels of high-density lipoprotein. The mechanism of AI extract’s anti-hyperglycemic effect was as an α-glucosidase inhibitor—a controller of postprandial hyperglycemia [16].

Although, in this study, the histopathological features of the pancreas in rats treated with *A. indica *root extract showed a lower percentage of fat cells than in the control group, statistical analysis did not result in a significant difference between the two groups. This study used ethanol extract from the root of *A. indica *with a dose of 230 mg/kg BW; previous studies used different parts and doses of *A. indica*. A survey of the anti-hyperlipidemia effects of *A. indica* showed that the ethanol extract was used from the leaves with a maximum effective dose of 400 mg/kg BW [11]. In comparison, a study on the anti-hyperglycemic postprandial effect of *A. indica* extract from all parts of the plant used a dose range of 300–600 mg/kg BW [14]. Both simvastatin and *A. indica* extract have anti-hyperlipidemia effects. In this study, there was a statistically significant difference in the percentage of the fatty pancreas in rats after being induced with a high fructose and cholesterol diet between the control and simvastatin–AI extract combination therapy group. However, there was no significant difference statistically. The percentage of fatty pancreas between the simvastatin–*A. indica *extract combination therapy group was lower than the simvastatin therapy group.

Simvastatin is a cytochrome enzyme (CYP) 3A4 substrate. Its plasma concentration will increase due to the inhibition of CYP3A4. Some flavonoids, such as quercetin and kaempferol, are inhibitors of the CYP3A4 enzyme, so their use in conjunction with simvastatin will increase the concentration of simvastatin in the plasma [17,18]. This may have a synergic effect and may also increase the risk of simvastatin side effects, such as rhabdomyolysis and myopathy. So, more research needs to be conducted to find out what kind of flavonoid is in *A. indica *extract and how it might interact with simvastatin.

## Conclusion

There was a significant difference between the percentage of the fatty pancreas in the control group and the simvastatin therapy group. The percentage of the fatty pancreas in the simvastatin–AI extract combination therapy group was lower than the simvastatin therapy group and the A. indica extract therapy group. Still, statistically, there was no significant difference.

## List of abbreviations

*Acalypha indica* root (AI), cytochrome enzyme (CYP), , low-density lipoprotein (LDL), nonalcoholic fatty pancreas disease (NAFPD).
